# 1,1′-(Ethane-1,2-di­yl)bis­(3-phenyl­thio­urea)

**DOI:** 10.1107/S1600536811039936

**Published:** 2011-10-05

**Authors:** Pramod B. Pansuriya, Holger B. Friedrich, Glenn E. M. Maguire

**Affiliations:** a School of Chemistry, University of KwaZulu-Natal, Durban 4000, South Africa

## Abstract

The complete molecule of the title compound, C_16_H_18_N_4_S_2_, is generated by crystallographic inversion symmetry. The dihedral angle between the phenyl ring and the thio­urea group is 52.9 (4)°. The crystal structure displays inter­molecular N—H⋯S hydrogen bonding, which generates sheets in the *ab* plane.

## Related literature

Bisthio­urea and urea derivatives with alkane bridges can adopt two general shapes, bent (Pansuriya *et al.*, 2011*a* ▶) or straight alkyl chains (Pansuriya *et al.*, 2011*b* ▶; Koevoets *et al.*, 2005 ▶). For the synthesis see: Lee *et al.* (1985 ▶).
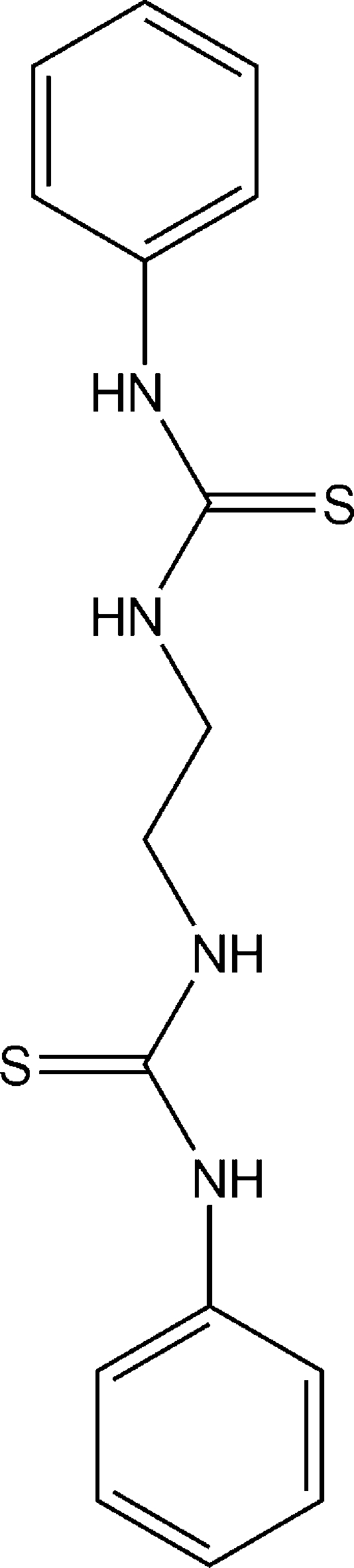


## Experimental

### 

#### Crystal data

C_16_H_18_N_4_S_2_
*M* *_r_* = 330.46
Orthorhombic, 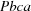

*a* = 10.5823 (4) Å
*b* = 9.1053 (3) Å
*c* = 16.4163 (6) Å
*V* = 1581.79 (10) Å^3^
*Z* = 4
Mo *K*α radiation
μ = 0.34 mm^−1^
*T* = 173 K
0.53 × 0.26 × 0.12 mm

#### Data collection

Bruker APEXII CCD diffractometer
22438 measured reflections
1902 independent reflections
1523 reflections with *I* > 2σ(*I*)
*R*_int_ = 0.078

#### Refinement

*R*[*F* ^2^ > 2σ(*F* ^2^)] = 0.054
*wR*(*F* ^2^) = 0.135
*S* = 1.13
1902 reflections
100 parameters
H-atom parameters constrained
Δρ_max_ = 0.55 e Å^−3^
Δρ_min_ = −0.38 e Å^−3^

### 

Data collection: *APEX2* (Bruker, 2006 ▶); cell refinement: *SAINT* (Bruker, 2006 ▶); data reduction: *SAINT*; program(s) used to solve structure: *SHELXS97* (Sheldrick, 2008 ▶); program(s) used to refine structure: *SHELXL97* (Sheldrick, 2008 ▶); molecular graphics: *OLEX2* (Dolomanov *et al.*, 2009 ▶); software used to prepare material for publication: *SHELXTL* (Sheldrick, 2008 ▶).

## Supplementary Material

Crystal structure: contains datablock(s) I, global. DOI: 10.1107/S1600536811039936/pk2349sup1.cif

Structure factors: contains datablock(s) I. DOI: 10.1107/S1600536811039936/pk2349Isup2.hkl

Supplementary material file. DOI: 10.1107/S1600536811039936/pk2349Isup3.cml

Additional supplementary materials:  crystallographic information; 3D view; checkCIF report

## Figures and Tables

**Table 1:** Hydrogen-bond geometry (Å, °)

*D*—H⋯*A*	*D*—H	H⋯*A*	*D*⋯*A*	*D*—H⋯*A*
N1—H1*N*⋯S1^i^	0.88	2.57	3.379 (2)	153

Symmetry code: (i) 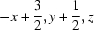.

**Fig. 1.**
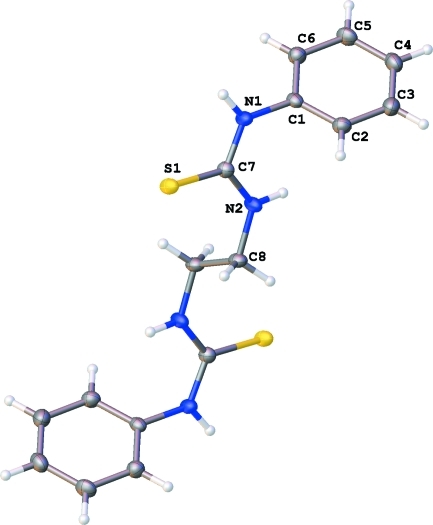
The molecular structure of the title compound. Displacement ellipsoids are drawn at the 40% probability level. The 1,1'-(ethane-1,2-diyl)bis(3-phenylthiourea) has inversion symmetry, so that unlabelled atoms are related by (1 - x, -y, 1 - z.

**Fig. 2.**
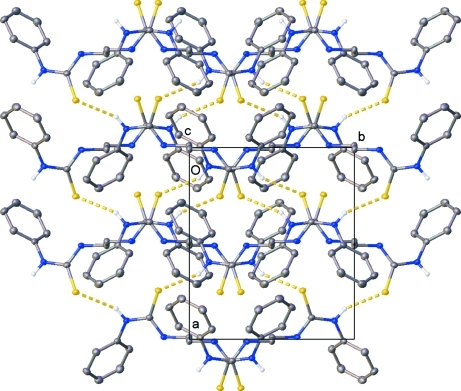
The crystal packing structure in the ab plane. All hydrogen atoms except those involved in hydrogen bonding interactions have been omitted for clarity.

## supplementary crystallographic information

### Comment

Thiourea and urea functionalized ligands play key roles in a wide range of catalytic reactions. Here we report the crystal structure of such a compound (Lee *et al.*, 1985) (Fig. 1). We recently reported a similar thiourea structure, where the molecules were bent (Pansuriya *et al.*, 2011*a*). Bisthiourea and urea derivatives with alkane bridges can adopt two general shapes, bent (Pansuriya *et al.*, 2011*a*) or straight alkyl chains (Pansuriya *et al.*, 2011*b*; Koevoets *et al.*, 2005). The spacer length between the two terminal thiourea or urea groups does not appear to influence the shape the bridging atoms take. The closest structure to the title compound 1,1'-(butane-1,4-diyl)bis(3-phenylthiourea) (Pansuriya *et al.*, 2011*a*) has also a *transoid* arrangement of the two thiourea groups. The asymmetric unit of the title compound is a half molecule and the complete molecule is generated by inversion symmetry (i): *1 - x, -y, 1 - z*. The structure shows intermolecular hydrogen bonding interactions between N1–H1···S1, 3.379 (2) Å, that creates sheets in the *ab* plane(Fig. 2). The dihedral angle between the phenyl ring and the thiourea group is 52.9 (4)°.

### Experimental

A solution of phenyl isothiocyanate (6.75 g, 50 mmol) in diethyl ether (15 ml) was added dropwise at 15 °C to a vigorously stirring solution of anhydrous ethane-1,2-diamine (6.01 g, 100 mmol) in isopropyl alcohol (100 ml) over a period of 30 min.  The reaction mixture was stirred for 2 hrs at room temperature and quenched with water (200 ml).  This reaction mixture was then maintained overnight at room temperature. Then the reaction mixture was acidified with conc. HCl up to pH 2.6. The solvents were evaporated under reduced pressure, the residue was suspended in hot water for 30 min. The resulting precipitate was filtered by vacuum. The product was washed with ice cold water and dried. The yield was 2.90 g (35%).

Crystals suitable for single-crystal X-ray diffraction analysis were grown in methanol: methylene chloride (1:2) at room temperature.  M.p. = 462 K.

### Refinement

Hydrogen atoms were first located in the difference map then positioned geometrically and allowed to ride on their respective parent atoms with C—H distances of 0.95 Å (C_ar_H), 0.99 Å (CH_2_) and N—H distances of 0.88 Å.  U_iso_(H) values were set to 1.2 U_eq_ of the attached atom.

### Crystal data

**Table:**

C_16_H_18_N_4_S_2_	*D*_x_ = 1.388 Mg m^−^^3^
*M**_r_* = 330.46	Melting point: 462 K
Orthorhombic, *P**b**c**a*	Mo *K*α radiation, λ = 0.71073 Å
Hall symbol: -P 2ac 2ab	Cell parameters from 8682 reflections
*a* = 10.5823 (4) Å	θ = 2.5–28.3°
*b* = 9.1053 (3) Å	µ = 0.34 mm^−^^1^
*c* = 16.4163 (6) Å	*T* = 173 K
*V* = 1581.79 (10) Å^3^	Plate, colourless
*Z* = 4	0.53 × 0.26 × 0.12 mm
*F*(000) = 696	

### Data collection

**Table:**

Bruker APEXII CCD diffractometer	1523 reflections with *I* > 2σ(*I*)
Radiation source: fine-focus sealed tube	*R*_int_ = 0.078
graphite	θ_max_ = 28.0°, θ_min_ = 2.5°
φ and ω scans	*h* = −13→13
22438 measured reflections	*k* = −12→12
1902 independent reflections	*l* = −21→21

### Refinement

**Table:**

Refinement on *F*^2^	Primary atom site location: structure-invariant direct methods
Least-squares matrix: full	Secondary atom site location: difference Fourier map
*R*[*F*^2^ > 2σ(*F*^2^)] = 0.054	Hydrogen site location: inferred from neighbouring sites
*wR*(*F*^2^) = 0.135	H-atom parameters constrained
*S* = 1.13	*w* = 1/[σ^2^(*F*_o_^2^) + (0.0373*P*)^2^ + 3.3624*P*] where *P* = (*F*_o_^2^ + 2*F*_c_^2^)/3
1902 reflections	(Δ/σ)_max_ < 0.001
100 parameters	Δρ_max_ = 0.55 e Å^−^^3^
0 restraints	Δρ_min_ = −0.38 e Å^−^^3^

### Special details

**Table:**

Geometry. All e.s.d.'s (except the e.s.d. in the dihedral angle between two l.s. planes) are estimated using the full covariance matrix. The cell e.s.d.'s are taken into account individually in the estimation of e.s.d.'s in distances, angles and torsion angles; correlations between e.s.d.'s in cell parameters are only used when they are defined by crystal symmetry. An approximate (isotropic) treatment of cell e.s.d.'s is used for estimating e.s.d.'s involving l.s. planes.
Refinement. Refinement of *F*^2^ against ALL reflections. The weighted *R*-factor *wR* and goodness of fit *S* are based on *F*^2^, conventional *R*-factors *R* are based on *F*, with *F* set to zero for negative *F*^2^. The threshold expression of *F*^2^ > σ(*F*^2^) is used only for calculating *R*-factors(gt) *etc*. and is not relevant to the choice of reflections for refinement. *R*-factors based on *F*^2^ are statistically about twice as large as those based on *F*, and *R*- factors based on ALL data will be even larger.

### Fractional atomic coordinates and isotropic or equivalent isotropic displacement parameters (Å^2^)

**Table:**

	*x*	*y*	*z*	*U*_iso_*/*U*_eq_	
C1	0.4907 (2)	0.4345 (3)	0.35565 (15)	0.0229 (5)	
C2	0.4488 (3)	0.3631 (3)	0.28604 (17)	0.0281 (6)	
H2	0.4897	0.2763	0.2678	0.034*	
C3	0.3463 (3)	0.4196 (3)	0.24308 (18)	0.0329 (6)	
H3	0.3161	0.3700	0.1961	0.039*	
C4	0.2885 (3)	0.5468 (3)	0.26835 (18)	0.0339 (6)	
H4	0.2185	0.5848	0.2389	0.041*	
C5	0.3321 (3)	0.6195 (3)	0.33647 (18)	0.0321 (6)	
H5	0.2932	0.7086	0.3530	0.039*	
C6	0.4330 (3)	0.5630 (3)	0.38118 (17)	0.0280 (6)	
H6	0.4619	0.6121	0.4287	0.034*	
C7	0.6127 (2)	0.2403 (3)	0.42665 (15)	0.0226 (5)	
C8	0.5090 (3)	0.0019 (3)	0.45415 (16)	0.0263 (5)	
H8A	0.5891	−0.0479	0.4398	0.032*	
H8B	0.4389	−0.0520	0.4276	0.032*	
N1	0.5975 (2)	0.3795 (2)	0.39925 (14)	0.0246 (5)	
H1N	0.6591	0.4418	0.4092	0.030*	
N2	0.5118 (2)	0.1523 (2)	0.42399 (14)	0.0258 (5)	
H2N	0.4421	0.1877	0.4025	0.031*	
S1	0.75428 (6)	0.18711 (7)	0.46353 (4)	0.0273 (2)	

### Atomic displacement parameters (Å^2^)

**Table:**

	*U*^11^	*U*^22^	*U*^33^	*U*^12^	*U*^13^	*U*^23^
C1	0.0182 (11)	0.0206 (12)	0.0298 (12)	−0.0016 (9)	−0.0006 (10)	0.0067 (10)
C2	0.0263 (13)	0.0241 (13)	0.0338 (13)	0.0016 (11)	0.0008 (11)	0.0000 (11)
C3	0.0346 (15)	0.0306 (14)	0.0335 (14)	−0.0042 (12)	−0.0079 (12)	0.0026 (12)
C4	0.0268 (14)	0.0340 (15)	0.0407 (15)	−0.0005 (12)	−0.0072 (12)	0.0107 (12)
C5	0.0238 (13)	0.0281 (14)	0.0445 (16)	0.0071 (11)	0.0010 (12)	0.0039 (12)
C6	0.0265 (13)	0.0266 (13)	0.0309 (13)	0.0009 (11)	−0.0005 (10)	−0.0005 (11)
C7	0.0170 (11)	0.0230 (12)	0.0279 (12)	0.0013 (9)	0.0023 (10)	0.0006 (10)
C8	0.0229 (12)	0.0184 (11)	0.0376 (14)	−0.0016 (9)	−0.0004 (11)	0.0030 (10)
N1	0.0164 (10)	0.0196 (10)	0.0378 (12)	−0.0019 (8)	−0.0033 (9)	0.0024 (9)
N2	0.0152 (10)	0.0219 (11)	0.0403 (12)	−0.0003 (8)	−0.0024 (9)	0.0084 (9)
S1	0.0150 (3)	0.0218 (3)	0.0450 (4)	0.0017 (2)	−0.0026 (3)	0.0001 (3)

### Geometric parameters (Å, °)

**Table:**

C1—C6	1.385 (4)	C6—H6	0.9500
C1—C2	1.387 (4)	C7—N2	1.336 (3)
C1—N1	1.428 (3)	C7—N1	1.354 (3)
C2—C3	1.393 (4)	C7—S1	1.687 (2)
C2—H2	0.9500	C8—N2	1.456 (3)
C3—C4	1.373 (4)	C8—C8^i^	1.518 (5)
C3—H3	0.9500	C8—H8A	0.9900
C4—C5	1.379 (4)	C8—H8B	0.9900
C4—H4	0.9500	N1—H1N	0.8800
C5—C6	1.394 (4)	N2—H2N	0.8800
C5—H5	0.9500		
			
C6—C1—C2	120.3 (2)	C5—C6—H6	120.3
C6—C1—N1	119.6 (2)	N2—C7—N1	117.1 (2)
C2—C1—N1	120.1 (2)	N2—C7—S1	123.3 (2)
C1—C2—C3	119.5 (3)	N1—C7—S1	119.59 (19)
C1—C2—H2	120.2	N2—C8—C8^i^	111.2 (3)
C3—C2—H2	120.2	N2—C8—H8A	109.4
C4—C3—C2	120.4 (3)	C8^i^—C8—H8A	109.4
C4—C3—H3	119.8	N2—C8—H8B	109.4
C2—C3—H3	119.8	C8^i^—C8—H8B	109.4
C3—C4—C5	120.0 (3)	H8A—C8—H8B	108.0
C3—C4—H4	120.0	C7—N1—C1	126.1 (2)
C5—C4—H4	120.0	C7—N1—H1N	117.0
C4—C5—C6	120.4 (3)	C1—N1—H1N	117.0
C4—C5—H5	119.8	C7—N2—C8	124.7 (2)
C6—C5—H5	119.8	C7—N2—H2N	117.6
C1—C6—C5	119.4 (3)	C8—N2—H2N	117.6
C1—C6—H6	120.3		
			
C6—C1—C2—C3	−1.5 (4)	N2—C7—N1—C1	−11.0 (4)
N1—C1—C2—C3	−178.6 (2)	S1—C7—N1—C1	170.1 (2)
C1—C2—C3—C4	1.3 (4)	C6—C1—N1—C7	130.0 (3)
C2—C3—C4—C5	0.1 (4)	C2—C1—N1—C7	−52.9 (4)
C3—C4—C5—C6	−1.4 (4)	N1—C7—N2—C8	−177.1 (2)
C2—C1—C6—C5	0.3 (4)	S1—C7—N2—C8	1.8 (4)
N1—C1—C6—C5	177.4 (2)	C8^i^—C8—N2—C7	80.6 (4)
C4—C5—C6—C1	1.2 (4)		

Symmetry codes: (i) −*x*+1, −*y*, −*z*+1.

### Hydrogen-bond geometry (Å, °)

**Table:**

*D*—H···*A*	*D*—H	H···*A*	*D*···*A*	*D*—H···*A*
N1—H1N···S1^ii^	0.88	2.57	3.379 (2)	153

Symmetry codes: (ii) −*x*+3/2, *y*+1/2, *z*.
